# sparrpowR: a flexible R package to estimate statistical power to identify spatial clustering of two groups and its application

**DOI:** 10.1186/s12942-021-00267-z

**Published:** 2021-03-18

**Authors:** Ian D. Buller, Derek W. Brown, Timothy A. Myers, Rena R. Jones, Mitchell J. Machiela

**Affiliations:** 1 Occupational and Environmental Epidemiology Branch, Division of Cancer Epidemiology and Genetics, National Cancer Institute, 9609 Medical Center Drive, Rockville, MD 20850 USA; 2 Cancer Prevention Fellowship Program, Division of Cancer Prevention, National Cancer Institute, Rockville, MD 20850 USA; 3 Integrative Tumor Epidemiology Branch, Division of Cancer Epidemiology and Genetics, National Cancer Institute, Rockville, MD 20850 USA; 4 Laboratory of Genetic Susceptibility, Division of Cancer Epidemiology and Genetics, National Cancer Institute, Rockville, MD 20850 USA

**Keywords:** Cancer incidence, Environmental epidemiology, Point pattern, Spatial clustering, Statistical power

## Abstract

**Background:**

Cancer epidemiology studies require sufficient power to assess spatial relationships between exposures and cancer incidence accurately. However, methods for power calculations of spatial statistics are complicated and underdeveloped, and therefore underutilized by investigators. The spatial relative risk function, a cluster detection technique that detects spatial clusters of point-level data for two groups (e.g., cancer cases and controls, two exposure groups), is a commonly used spatial statistic but does not have a readily available power calculation for study design.

**Results:**

We developed *sparrpowR* as an open-source R package to estimate the statistical power of the spatial relative risk function. *sparrpowR* generates simulated data applying user-defined parameters (e.g., sample size, locations) to detect spatial clusters with high statistical power. We present applications of *sparrpowR* that perform a power calculation for a study designed to detect a spatial cluster of incident cancer in relation to a point source of numerous environmental emissions. The conducted power calculations demonstrate the functionality and utility of *sparrpowR* to calculate the local power for spatial cluster detection.

**Conclusions:**

*sparrpowR* improves the current capacity of investigators to calculate the statistical power of spatial clusters, which assists in designing more efficient studies. This newly developed R package addresses a critically underdeveloped gap in cancer epidemiology by estimating statistical power for a common spatial cluster detection technique.

## Background

Geospatial study approaches are used to investigate the location of incident cancer cases in relation to potential sources of known or suspected environmental carcinogens (e.g., pesticides, industrial air emissions). By using specific locations of study participant residences (i.e., point locations), cancer incidence can be described, from a spatial perspective, utilizing spatial point pattern processes and further evaluated with statistical functions. The spatial relative risk (SRR) function is widely utilized to determine where detected spatial clustering is likely occurring (i.e., local clustering test statistic; 1–3) Originally designed to study the spatial variation of larynx and lung cancers in Lancashire, United Kingdom in relation to proximity to an industrial incinerator [2, 3], the SRR function has been applied to detect clustering in many other epidemiologic investigations of cancer such as childhood leukemia in Ohio [4], late-stage colorectal cancer in Iowa [5], and breast cancer in New York [6]. Geospatial approaches can improve investigations of cancer etiology, but the challenge is designing a study with adequate power to detect real spatial clusters (versus a statistical artifact) and whether spatially distributed exposures can explain it.

Within the context of study design, statistical power is used to determine if a proposed study can yield valid inferences. Well-designed spatial studies are of paramount importance, as those conducted with low power will use limited resources and time and will likely produce insignificant p-values and poor precision in effect estimates [7, 8]. Although many online tools and statistical software can directly calculate statistical power given study parameters (e.g., disease prevalence, effect size, sample size), these calculations are not adequate for spatial study designs as they are often oversimplified and ignore fundamental assumptions of spatial analyses (e.g., spatial autocorrelation). While the SRR function has been highly utilized across many diseases/spatial analyses, there is currently no available power calculation for its local clustering statistic. None of the aforementioned investigations include a discussion of statistical power to detect local clustering [1–6].

We developed *sparrpowR* as an open-source R statistical programming package [9] to calculate statistical power for the local statistic of the SRR function [1–3] using simulation-based techniques. *sparrpowR* utilizes available R [10] functionality to generate reproducible spatially clustered point-level data and further detects areas with highly powered spatial clusters within two groups (e.g., cancer case and non-cancer control locations, or two exposure groups). Our R package [9] will enable a more efficient and appropriate design and analysis of future environmental epidemiologic studies, increasing both the quality and impact of spatial studies. We present an application of *sparrpowR* to perform power calculations for two epidemiologic study designs. This application details both the flexibility and useability of the tool and further demonstrates *sparrpowR*’s capability to determine necessary sample sizes when designing a study.

## Methods

### Power calculation algorithm

*sparrpowR* calculates statistical power for the local statistic of the SRR function [1–3] to identify highly powered spatial clustering of one group relative to another. Briefly, the SRR function compares the pattern of two groupings of point locations (e.g., patients with cancer versus community controls) with the ratio of their bivariate (e.g., latitude and longitude) densities that are smoothed into a gridded surface of *z* locations:

1 $$r\left(z\right)=\frac{f\left(z\right)}{g\left(z\right)},$$

where $f$ is the bivariate probability density of the geographical coordinates of cases of the disease across the study area and $g$ is the density of controls over the same region [1–3]. The SRR function (Eq. 1) is commonly presented as the natural logarithm transformed log-relative risk function $\rho (z)= \mathrm{log}(r\left(z\right))$. The function does not incorporate covariates, only the spatial densities of the two groups. The SRR function was recently extended to estimate the knot (i.e., grid cell) in which the observed density of cases exceeds a null asymptotic normal expectation [11]; the null hypothesis of which is no spatial clustering of one group relative to another [2] and the alternative hypothesis where such clustering is present:

2a $${H}_{0}:\uprho \left(\mathrm{z}\right)=0$$

2b $${H}_{A}:\uprho \left(\mathrm{z}\right)\ne 0.$$

*sparrpowR* utilizes built-in *R* [10] functionality using the *sparr* package [12] to calculate the SRR function, including default parameters for bandwidth (maximal smoothing principle [13]) and resolution (128 × 128 grid) that can also be user-specified if desired. *sparrpowR* also utilizes built-in *R* [10] functionality to generate reproducible spatially clustered data that reflect an expected study design. In particular, spatial data is simulated after a user specifies the number of expected clusters, and points may be generated such that they concentrate in certain areas (i.e., around exposure point sources) to reflect an expected prevalence of exposure. *sparrpowR* [10] can simulate several spatial distributions including, but not limited to, complete spatial randomness, uniform, and multivariate normal distributions using functionality from the *spatstat* package [14]. For example, the multivariate normal distribution for a simulated two-dimensional location $i$ with coordinates ${(x}_{i},{y}_{i})$ is a random normal distance in each dimension from a center point with coordinates ${(x}_{0},{y}_{0})$ based on a defined standard deviation $(\sigma )$ and mean zero:

3a $${x}_{i}= {x}_{0}+\mathrm{N}(0,\upsigma )$$

3b $${y}_{i}= {y}_{0}+\mathrm{N}\left(0,\upsigma \right).$$

Further user-defined parameters (e.g., disease prevalence, total sample size, detection area) give *sparrpowR* the flexibility to generate a wide variety of clustering data.

Power calculations within *sparrpowR* involve randomly simulating data that reflect expected sampling for the desired study and performing realistic spatial analyses [15, 16]. Although simulation-based procedures may be computationally intensive, as simulations are repeated often (e.g., 10,000 iterations), study power derived in this manner is more reliable as it represents real data [15, 16]. Recent improvements to the SRR function [11, 12, 17], namely the asymptotic normality approximation for the hypothesis testing (Eqs. 2a and 2b), make the proposed simulation-based power calculation method feasible. The following steps detail the power calculation procedure utilized within *sparrpowR*:

1. Generate point-level data based on investigator-defined inputs that reflect the expected study design (see Baddeley et al. for a detailed discussion of simulated point-pattern data [14]).
2. Calculate the SRR function for each knot (i.e., grid cell) within the simulated data area.
3. Retain the significance status (yes/no) of observed spatial clustering of each knot at a given alpha level.
4. Repeat steps 1–3 for 10,000 iterations (user-specified) by generating new data under the same user-defined parameters within each iteration to create a set of associated decisions of statistical significance. Within each iteration, the control locations are re-simulated to provide a new control distribution following the same parameters in Step 1, on which the SRR function is recalculated (Step 2). Importantly, the case locations are simulated once in the first iteration, and the exact same case distribution is used in all subsequent iterations.
5. Record the number of simulations in which the null hypothesis is rejected.
6. At each knot, calculate statistical power as the proportion of rejected null hypotheses from the set of simulations noted in step 5 to give the final local power results.

The power calculation output is a local spatial measure (i.e., at each grid cell), not a global spatial measure (i.e., across the entire study area), which identifies local zones within a study area that are sufficiently powered to detect spatial clustering.

The *sparrpowR* package [9] is self-containing and provides functions to simulate data (spatial_data), calculate statistical power (spatial_power), and visualize the data inputs and outputs (spatial_plots). Other examples and additional information about computing efficiency and parameter selection for the *sparrpowR* package are available in the vignette on the Comprehensive R Archive Network [9].

### Data application #1: surveillance

To demonstrate the utility of *sparrpowR* to calculate the power for the local statistic of the SRR function, we conducted an example surveillance-based power calculation for the detection of spatial clusters of non-Hodgkin lymphoma (NHL) cases in relation to a concentrated animal feeding operation (CAFO), a point source for numerous environmental emissions [18]. The purpose of this example power calculation was to determine if a surveillance study is sufficiently powered to detect an observed spatial cluster of incident NHL cases near the CAFO within a prospective cancer cohort.

#### NHL cases

A recent study by Fisher et al*.* found an association between NHL incidence in Iowa farmers and the intensity of animal production from CAFOs within 5 km of their residence [19]. Here, we used a population-based prospective cohort of postmenopausal women in the Iowa Women’s Health Study (IWHS; enrolled in 1986 with follow-up for cancer incidence through 2009; 18) to compute the SRR function (analogous to a case–control comparison) for 8 incident NHL cases identified within 5 km of an Environmental Protection Agency-defined medium-sized CAFO (> 800 animal units; 19) in Fort Dodge, Iowa from the Iowa Department of Natural Resources [22]. Within the given study window around Fort Dodge, the IWHS enrolled 436 women, which equates to a study incidence of NHL of 1,834.9 per 100,000, almost 80-fold larger than the estimated 2009 U.S. national incidence of NHL (20.6 per 100,000 [23]). Based on the elevated incidences of NHL in the study area and study parameters, we wanted to determine if the study in the IWHS is sufficiently powered to detect true spatial clusters of incident NHL cases. To protect personally identifying information, we did not use the “true” locations of the NHL cases from IWHS. Instead, we used a single simulation to set the location of 8 NHL cases assuming a multivariate normal (MVN) distribution with a standard deviation of 0.83 km centered at the identified CAFO.

#### Simulated controls and power calculation

**Fig. 1:**
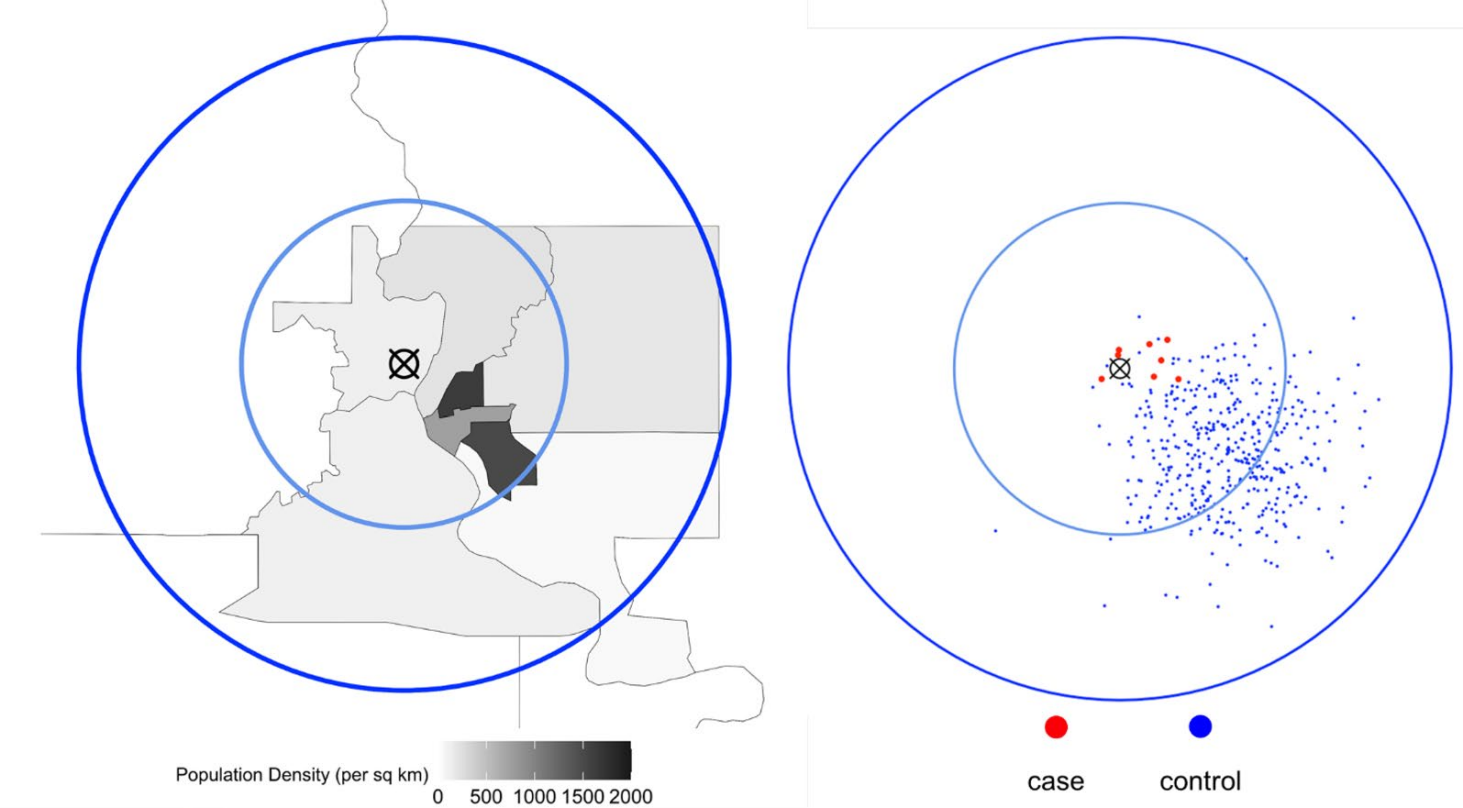
Comparison between the census tract of Fort Dodge, Iowa (Left) and the first iteration of simulated data (Right). Using *sparrpowR*, 8 cases and 428 controls were generated assuming multivariate normal distributions with standard deviations of 0.83 and 1.67 km, respectively. Simulated case locations are red-colored dots and simulated control locations are blue-colored dots. The identified concentrated animal feeding operation (CAFO) is signified by the black “X” symbol. The blue lines represent regions with radii of 5 and 10 km from the identified CAFO

To conduct power calculations based on the population density of Fort Dodge, Iowa, we used population estimates from the 2010 U.S. Decennial Census in census tracts 10 km from our identified CAFO [24]. Based on the population density within the study window (Fig. 1), we simulated controls assuming an MVN distribution with a standard deviation of 1.67 km centered at Fort Dodge, Iowa (Fig. 1). Based on this sampling scheme, it is clear that the simulated control locations reflect the true population density around the identified CAFO.

We simulated 10,000 random iterations using an overall sample size of 436 (8 cases and 428 controls) and calculated the statistical power for the local statistic of the SRR function using the *sparrpowR* package [9]. We used the default alpha level (0.05, two-tailed) and power threshold (0.8) within the power calculation. We performed a sensitivity analysis of increased sample size to demonstrate the functionality of *sparrpowR*, holding all other study parameters constant. The larger sample size was 1,000 (18 cases and 982 controls, keeping the NHL incidence within 10 km of the identified CAFO of 1,834.9 per 100,000 constant).

### Data application #2: etiology

The *sparrpowR* package may also be used to conduct etiologic-based power calculations. These calculations are used to inform study design as they help answer questions related to the number of samples needed to have a sufficiently powered study of the association between environmental exposures and a disease outcome. We conducted six additional simulation scenarios with various incidence rates and sample sizes to further demonstrate the utility of *sparrpowR*. We performed new power calculations within the Fort Dodge, Iowa area using the same sampling methods and parameters as the previous calculations updating the incidence rate and sample size (Additional file 1: Table S1). Incidence rates ranged from the U.S. national rate of NHL (20.6 per 100,000) to the NHL incidence within 10 km of the identified CAFO (1834.9 per 100,000) under two sample sizes, 10,000 and 24,000 (approximately the total 2010 population of Fort Dodge, Iowa).

All statistical code for the two data applications is available in the Online Code Repository and can be used to replicate our results fully.

## Results

**Fig. 2:**
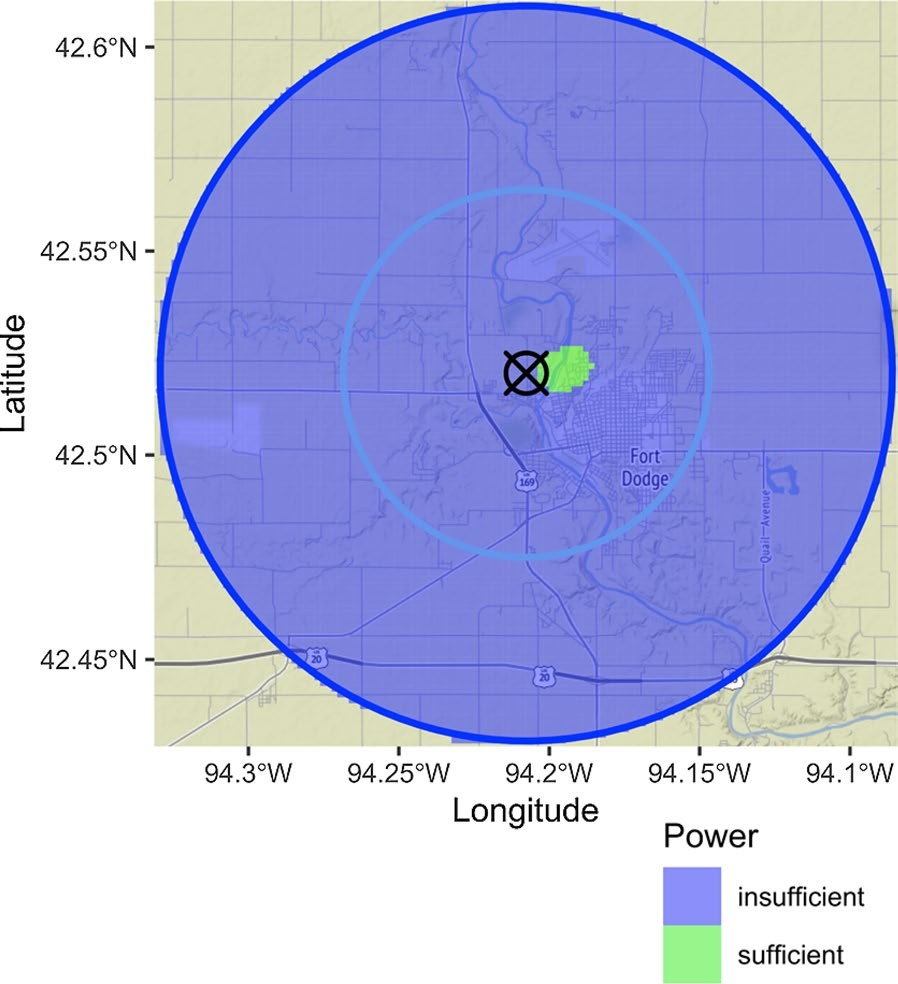
Results of 10,000 *sparrpowR* iterations simulating 8 cases and 428 controls assuming multivariate normal distributions with standard deviations of 0.83 and 1.67 km, respectively. The green-colored area is sufficiently powered to detect a spatial cluster of cases relative to controls. The blue-colored area is insufficiently powered to detect a spatial cluster of cases relative to controls. The identified concentrated animal feeding operation (CAFO) is signified by the black “X” and the base map is of Fort Dodge, Iowa. The blue lines represent regions with radii of 5 and 10 km from the identified CAFO

**Fig. 3:**
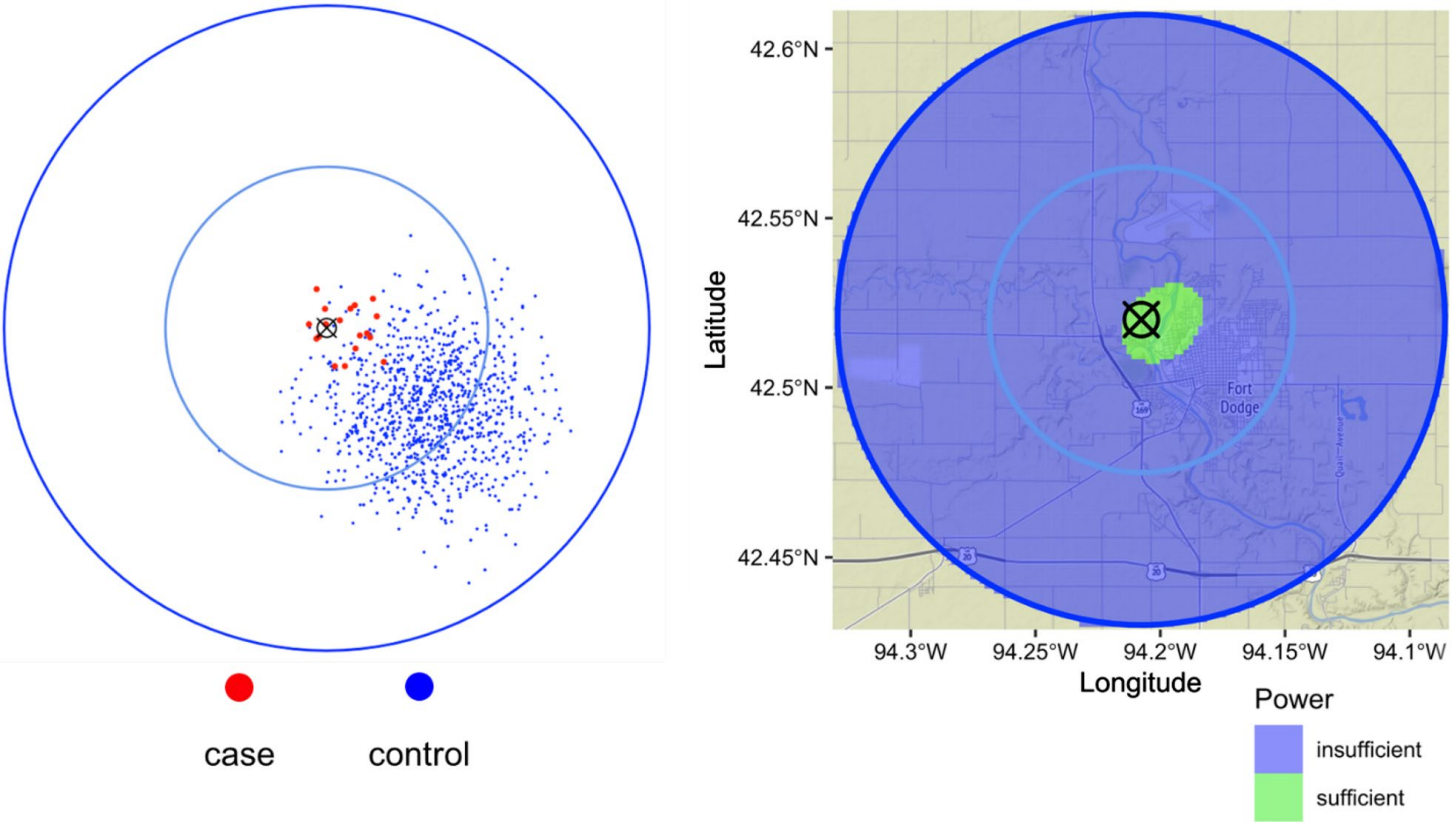
Results of 10,000 *sparrpowR* iterations simulating 18 cases and 982 controls assuming multivariate normal distributions with standard deviations of 0.83 and 1.67 km, respectively. (Left) The first iteration of simulated data. Simulated case locations are red-colored dots and simulated control locations are blue-colored dots. (Right) The green-colored area is sufficiently powered to detect a spatial cluster of cases relative to controls. The blue-colored area is insufficiently powered to detect a spatial cluster of cases relative to controls. The identified concentrated animal feeding operation (CAFO) is signified by the black “X” and the base map is of Fort Dodge, Iowa. The blue lines represent regions with radii of 5 and 10 km from the identified CAFO

Based on the given study parameters for the first data application, we were sufficiently powered to detect one small spatial cluster of NHL cases (relative to control locations) to the east of the identified CAFO (Fig. 2). This result indicates that the study is well powered to detect a spatial cluster of incident NHL cases surrounding a CAFO in the IWHS. When we increased the overall sample size, the identified sufficiently powered zone (Fig. 3) was larger than the one detected using the smaller sample size from the IWHS (Fig. 2).

For the second data application, as both incidence rate and sample size increased, the sufficiently powered area to detect an NHL cluster around an environmental exposure also increased (Additional file 1: Figure S1). Given the U.S incidence rate, sampling the entire population of Fort Dodge, Iowa does not lead to a well-powered study (Additional file 1: Figure S1b) while using an incidence rate half of the NHL incidence within 10 km of the identified CAFO, still produces sufficiently powered study areas (Additional file 1: Figure S1c, d).

## Discussion

*sparrpowR* is an open-source R statistical package [9] that improves upon a well-established geospatial technique by further providing epidemiologists a method to calculate its local statistical power, facilitating the design of robust geospatial studies. Without statistical power calculations, studies may have low power to determine where a cancer cluster is located and may unknowingly draw spurious conclusions about an association between cancer incidence and environmental exposures under investigation. This tool will have an impact not only on environmental cancer epidemiology but also on any discipline focused on detecting relative spatial clusters of point-level data. For example, *sparrpowR* could be used when designing spatial investigations of infectious diseases, geographic distributions of animal species, geo-tagged financial information, or any study that plans on utilizing the SRR function to detect the presence of spatial clusters between two groups.

The strength of the SRR function has been driven by its nonparametric flexibility to detect spatial clusters (i.e., clusters not limited to ellipsoids) [11], but this flexibility presents challenges for calculating the power of the local statistic. Our NHL power calculation is sensitive to the sample size and expected sampling distribution of case and control groups, and the size of the study area. In practice, power calculations should be conducted with realistic sampling strategies and sample sizes to produce well-designed spatial studies. Future sensitivity analyses using *sparrpowR* are warranted to determine the most influential factors when conducting spatial power calculations. Additionally, *sparrpowR* calculates the power for only one spatial statistic, the SRR function. Although there are power calculations available for other spatial statistics such as, for example, Moran’s *I* and Cuzick-Edwards [25], we present the first readily available power calculation for the local statistic of the SRR function [1–3]. Future functionality for *sparrpowR* includes simulating non-point exposures (e.g., linear network of roads as a source of air pollution) and multiple testing correction options.

## Conclusions

Overall, *sparrpowR* addresses a critically underdeveloped gap in spatial epidemiology studies by providing an easy-to-implement method to calculate the statistical power for spatial cluster detection using the SRR function. Associations from studies that utilize our tool can be directly implemented into public health practice to improve surveillance and etiologic studies.

## Supplementary Information

**Additonal file 1: Table S1:** Supplemental Figure simulation scenario parameters. **Figure S1.** Results using 10,000 *sparrpowR* iterations simulating six scenarios with changing incidence and sample size, detailed in Table S1. Each scenario was conducted assuming multivariate normal distributions for cases and controls with standard deviations of 0.83 and 1.67 km, respectively. The green-colored areas are sufficiently powered to detect spatial clusters of cases relative to controls. The blue-colored areas are insufficiently powered to detect spatial clusters of cases relative to controls. The identified concentrated animal feeding operation (CAFO) is signified by the black “X” and the base map is of Fort Dodge, Iowa. The blue lines represent regions with radii of 5 and 10 km from the identified CAFO.

## Data Availability

Project name: sparrpowR. Project home page: https://github.com/machiela-lab/sparrpowR. Archived version: https://CRAN.R-project.org/package=sparrpowR. Operating system: Platform independent. Programming language: R. Other requirements: R 3.5.0 or higher. License: Apache License 2.0.

## Abbreviations

CAFO: Concentrated animal feeding operation
IWHS: Iowa Women’s Health Study
MVN: Multivariate normal
NHL: Non-Hodgkin lymphoma
SRR: Spatial relative risk
