# Supplementary material for: sparrpowR: a flexible R package to estimate statistical power to identify spatial clustering of two groups and its application
Source: Int J Health Geogr. 2021 Mar 18;20:13. doi: 10.1186/s12942-021-00267-z (PMC7977178; doi:10.1186/s12942-021-00267-z)
Supplement: Supplementary file 1 — Additonal file 1: Table S1: Supplemental Figure simulation scenario parameters. Figure S1. Results using 10,000 sparrpowR iterations simulating six scenarios with changing incidence and sample size, detailed in Table S1. Each scenario was conducted assuming multivariate normal distributions for cases and controls with standard deviations of 0.83 and 1.67 km, respectively. The green-colored areas are sufficiently powered to detect spatial clusters of cases relative to controls. The blue-colored areas are insufficiently powered to detect spatial clusters of cases relative to controls. The identified concentrated animal feeding operation (CAFO) is signified by the black “X” and the base map is of Fort Dodge, Iowa. The blue lines represent regions with radii of 5 and 10 km from the identified CAFO. [file 12942_2021_267_MOESM1_ESM.docx]

**Additional Material**

| **Supplement Table 1**. Supplemental Figure simulation scenario parameters | | |
| --- | --- | --- |
| Figure | Incidence (per 100,000) | N |
| A | 20.6 | 10,000 |
| B | 20.6 | 24,000 |
| C | 917.4 | 10,000 |
| D | 917.4 | 24,000 |
| E | 1,834.9 | 10,000 |
| F | 1,834.9 | 24,000 |


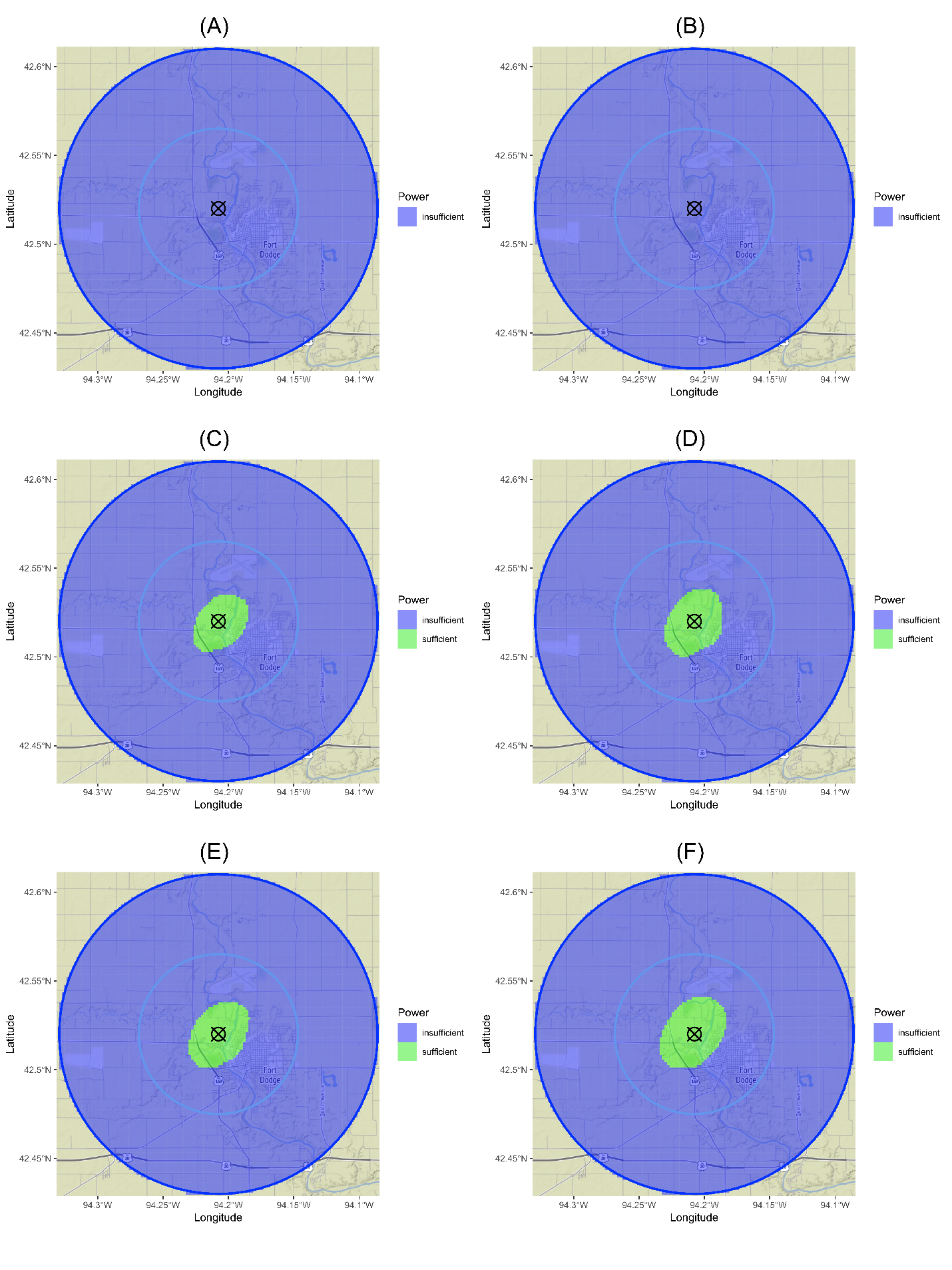


**Supplemental Figure 1:** Results using 10,000 *sparrpowR*iterations simulating six scenarios with changing incidence and sample size, detailed in **Supplemental Table 1**. Each scenario was conducted assuming multivariate normal distributions for cases and controls with standard deviations of 0.83 and 1.67 kilometers, respectively. The green-colored areas are sufficiently powered to detect spatial clusters of cases relative to controls. The blue-colored areas are insufficiently powered to detect spatial clusters of cases relative to controls. The identified concentrated animal feeding operation (CAFO) is signified by the black “X” and the base map is of Fort Dodge, Iowa. The blue lines represent regions with radii of 5 and 10 kilometers from the identified CAFO.
